# Coping with adverse childhood experiences during the COVID-19 pandemic: Perceptions of mental health service providers

**DOI:** 10.3389/fpsyg.2022.975300

**Published:** 2022-09-08

**Authors:** Sumaita Choudhury, Paul G. Yeh, Christine M. Markham

**Affiliations:** ^1^Department of Health Promotion and Behavioral Sciences, UTHealth School of Public Health, The University of Texas Health Science Center at Houston School of Public Health, Houston, TX, United States; ^2^Department of Health Promotion and Behavioral Sciences, UTHealth School of Public Health, The University of Texas Health Science Center at Houston School of Public Health, Brownsville, TX, United States

**Keywords:** adverse childhood experience, COVID-19, mental health services, trauma-informed therapy, resilience, social connectedness

## Abstract

**Background:**

Adverse Childhood Experiences (ACEs) have been associated with long-term physical and mental health conditions, toxic stress levels, developing unstable interpersonal relationships, and substance use disorders due to unresolved childhood adversities.

**Aims:**

This study assessed the perspectives of mental health providers (MHPs) regarding their adult patients’ coping with ACEs during COVID-19 in Houston, Texas. Specifically, we explored how individuals with ACEs are coping with the increased stresses of the pandemic, how MHPs may provide therapeutic support for individuals with ACEs during this pandemic, pandemic-related challenges of accessing and utilizing mental health services for individuals with ACEs, and the awareness and treatment of ACEs among MHPs.

**Methods:**

Ten in-depth semi-structured virtual interviews were conducted with licensed MHPs from November 2021 to April 2022 in Houston, Texas. Interviews were coded and analyzed for emerging themes through an inductive open coding approach to discover insights regarding coping with ACEs during COVID-19.

**Results:**

Four key themes experienced by individuals with ACEs emerged from the MHP interviews: (1) Maladaptive emotional dissonance and coping outlets during the pandemic, (2) Difficulties with social connectedness and significance of social support, (3) Heightened daily life stressors and coping with the ongoing disruption of the pandemic, and (4) Changing interactions with the mental health system. Themes from this study highlighted that resilience, seeking treatment, and strong social support can help develop healthy coping strategies among individuals with ACEs.

**Conclusion:**

This study may help inform best clinical practices to develop interventions and policies regarding ACEs such as a resilience-promotion approach that targets all the socio-ecological levels. In addition, findings highlight the synergy of psychotherapeutic and pharmacological management *via* tele-health modalities, in helping individuals with ACEs continue receiving the care they deserve and need during a persistent pandemic and an uncertain future.

## Introduction

According to the Centers for Disease Control and Prevention (CDC), adverse childhood experiences (ACEs) are traumatic events outside the realm of typical childhood developmental experiences that occur during childhood (Centers for Disease Control and Prevention [CDC], 2020). ACEs include traumatic events such as sexual and emotional violence, parental neglect, financial hardships, household dysfunction, and loss of family members (Centers for Disease Control and Prevention [CDC], 2020). Epidemiologically, the Centers for Disease Control and Prevention (2019) has reported that approximately 61.5% of adults have been exposed to ACEs in the United States. ACEs have been associated with long-term chronic physical and mental health conditions, toxic stress levels, developing unhealthy interpersonal relationships, and substance use disorders among adults due to psychologically unresolved childhood adversities (Brockie et al., 2015; Chanlongbutra et al., 2018; Bethell et al., 2019; Centers for Disease Control and Prevention [CDC], 2020; Doom et al., 2021).

In 1998, the CDC and Kaiser Permanente published the first study to examine ACE-related health outcomes and behaviors and found that individuals with multiple ACEs had poorer health outcomes, with a greater risk of all-cause mortality and medical comorbidities, such as coronary heart disease and autoimmune disorders (Mersky et al., 2013). Beyond the physical health impacts of ACE exposure lies the development of mental health disorders, including anxiety, depression, and an increased likelihood of illicit substance use disorders (Mersky et al., 2013). High-stress levels stemming from the trauma experiences of exposure to ACEs may precipitate emotional difficulties among adults, leading to struggles maintaining healthy relationships, unstable employment, and increased distrustfulness of others (Woods-Jaeger et al., 2018; Centers for Disease Control and Prevention [CDC], 2020). Furthermore, a dose-response relationship exists between increased ACEs and increased prevalence of suicide attempts, post-traumatic stress disorder (PTSD), substance use disorders, and risky sexual behaviors (Dube et al., 2001; Brockie et al., 2015; Chanlongbutra et al., 2018; Petruccelli et al., 2019). Thus, preventive interventions regarding ACEs must be developed, and individuals should be routinely screened and treated for their ACEs by their healthcare providers to decrease the risks of maladaptive coping behaviors (Dube et al., 2001; Monnat and Chandler, 2015).

Although the long-term adverse health consequences of ACEs have been studied, there has been relatively limited research regarding the protective social and community factors that can attenuate the association between poor health outcomes and adversity among individuals (Liu et al., 2019). Individuals with protective factors, such as access to trusted adults and continuous emotional and mental support from their families, schools, or communities, had better health outcomes (Bellis et al., 2017; Liu et al., 2019). Conversely, adults with increased ACE exposure levels who did not have continuous support from trusted adults reported a higher prevalence of risky behavioral patterns, including unhealthy dietary habits, substance use disorders, and poor mental health (Bellis et al., 2017). Protective factors to mitigate ACE health impacts include promoting resilience through trauma-informed approaches and a healthier lifestyle, enhancing supportive relationships, and augmenting social connectedness (Sacks and Murphey, 2018). Moreover, providers should help individuals recognize the impact of trauma from ACEs, address ACEs symptoms, and help to develop interventions to prevent further trauma among individuals with ACEs (Sacks and Murphey, 2018). Specifically, individual trauma-informed psychotherapy treatments such as cognitive behavioral therapy (CBT), dialectical behavioral therapy (DBT), prolonged exposure therapy (PE) have been effective in helping patients with ACEs develop positive coping strategies to regulate negative emotions and reduce ACEs’ long-term consequences (Di Lemma et al., 2019).

The ongoing COVID-19 pandemic policies of lockdowns and quarantining have made it difficult for individuals with ACEs to maintain positive intrapersonal coping mechanism skills. Simultaneously, individuals have experienced increased stress and anxiety due to accentuated exposure to potential domestic abuse or neglect at home, and limited interpersonal social support, which is critical for recovery from ACE symptoms (Bledsoe et al., 2021). Furthermore, the transition to telehealth therapy appointments during the pandemic facilitated healthcare access to mental health providers (MHPs) among some individuals with ACEs but, on the other hand, made it extremely difficult for populations with low levels of digital literacy or access to adequate digital technology to successfully access virtual health services (Bledsoe et al., 2021). Although there has been ample research regarding the impact of ACEs, less is known regarding how patients are coping with ACEs in the context of the heightened stress and disruption of the COVID-19 pandemic. MHPs who provided trauma-informed psychotherapy treatments regarding ACEs during the COVID-19 pandemic are an excellent source to disseminate significant information about ACE coping strategies and additional related barriers encountered during the pandemic.

Accordingly, in this qualitative study, we assessed the perspectives of MHPs regarding their adult patients’ coping with ACEs during COVID-19 in Houston, Texas. This study may help inform timely and relevant efforts during a persistent pandemic and an uncertain future to develop interventions. This study explored how individuals with ACEs are coping with the increased stresses of the pandemic, how MHPs could provide therapeutic support for individuals with ACEs during this pandemic, the pandemic-related challenges of accessing mental health services for individuals with ACEs, and the awareness and treatment of ACEs among MHPs.

## Materials and methods

### Design and setting

We conducted in-depth semi-structured virtual interviews with ten licensed MHPs from November 2021 to April 2022. Participants who were currently licensed MHPs in Houston, Texas (i.e., psychologists, psychiatrists, social workers, and counselors) and provided services related to ACEs and trauma to adult patients during the COVID-19 pandemic were recruited to participate in the study. Initially, recruitment was conducted using convenience sampling through social media channels (i.e., Facebook and LinkedIn) but this approach yielded insufficient eligible participants. Thus, both purposive and snowball sampling techniques were used for recruiting licensed MHPs and were employed through targeted emails and phone calls. No incentives were provided to the MHPs to participate in this study. We used a semi-structured interview guide with 10 broad questions and additional probe questions. The semi-structured interview guide was developed following an established interview structure (Tracy, 2020).

The interview guide followed a chronological order beginning with opening questions, including asking for verbal consent. Opening questions regarding the average number of patients they saw on a weekly basis and how long they had been practicing as MHPs provided context related to their perspective on treating patients with ACEs. Experience questions explored shared experiences with ACEs among their patients; positive coping mechanisms to combat symptoms related to ACEs from their patients; the impact of COVID-19 on getting mental health services for their patients; factors that helped or made it difficult to cope with ACEs; the role of religion, culture, and spirituality regarding coping with ACEs, and how ACEs affected their racially/ethnic minority patients specifically. Finally, catch-all questions were used to close the interview, which asked participants about their perspective on the long-term effects of COVID-19 on coping with ACEs, treatments that have been helpful in addressing the trauma related to ACEs, and provided space for participants to share any additional information they felt was relevant but had not been addressed (Tracy, 2020). The semi-structured interview guide is presented in Table 1. The study was reviewed and approved by the authors’ Institutional Review Board at the University of Texas Health Science Center at Houston.

**TABLE 1:** Semi-structured interview guide.

Interview questions	Purpose
**Opening Questions**	
1. Tell me about your practice as a psychologist, a psychiatrist, or a counselor?	• Initiate the interview
a. How many clients on average get referred to you?	
**Experience Questions**	
2. Tell me about ACEs in your adult patient population? I’m particularly interested in your patients’ experiences.	• Initiate the interview
a. What sort of impact do you think these experiences will have on your patients’ futures?	• Help participants get comfortable to start talking
3. How have ACEs among your adult patient population affected their lives?	• Help participant talk openly about the topic in an exploratory way
4. What are the most common stories that you have seen among your patients due to ACEs?	• Help participant talk openly about the topic in an exploratory way
	• Obtain greater detail about the responses
5. How has COVID-19 affected your adult patient population?	• Answer the research question(s)
a. How has COVID-19 affected your adult patient population in getting mental health services for their ACEs?	• Obtain greater detail about the responses
b. How have COVID-related events such as lockdown and quarantining affected your adult patient population in coping with their ACEs?	
c. How have different waves of COVID-19 cases and death rates affected your adult patient population in coping with their ACEs?	
d. How has the implementation of COVID-19 vaccines impacted you patients with ACEs in seeking mental health services?	
6. Based on your experience, tell me about different factors that have helped your adult patient population cope with ACEs during COVID-19 and in general?	• Help participant talk openly about the topic in an exploratory way
	• Obtain greater detail about the responses
7. Based on your experience, tell me about different factors that have made it hard for your adult patient population to cope with ACEs in their everyday lives during COVID-19 and in general?	• Help participant talk openly about the topic in an exploratory way
	• Obtain greater detail about the responses
8. Based on your observations, how has spirituality played a role in coping with ACEs among your adult patient population, especially during COVID-19?	• Obtain greater detail about the responses
	• Help participant talk openly about the topic in an exploratory way
**Catch-All Questions**	
9. Based on your observations, what kind of long-term effects do you think COVID-19 will have on your adult patient population coping with ACEs and getting mental health services?	• Answer the research question(s)
10. Based on your observations, what other factors have impacted your adult patient population with ACEs in getting mental health services?	• Answer the research question(s)
a. What kind of treatments has been effective among your adult patient population to cope with their ACEs?	
b. How do you think a person with ACEs can live their life positively?	
11. Is there anything else, since I’m not as familiar with this world as you, that could help me understand individuals living with ACEs, mental health services they receive, and the impact of COVID on these services?	• Wrap up

### Data collection

The first author conducted all interviews virtually *via* Zoom in a private room at a scheduled date preferred by the study participants. The interviews lasted approximately 30 min to an hour. The first author obtained verbal consent from each MHP before initiating the interview. The interviews were audio-recorded and transcribed verbatim using Temi audio to text transcription services.^1^ After the interviews were conducted, the researcher took field notes to record important observations regarding each interview. Using the grounded theory approach, MHPs were recruited until data saturation was reached and no new evidence was provided. All interview transcriptions, notes, audio, and video recordings were stored in a password-protected computer system, which was only accessible to the research team.

### Qualitative analysis

The interviews were coded and analyzed using the qualitative data analysis software, NVivo student software version 12.0. The first author reviewed all the interview transcripts and developed initial codes using an inductive open coding approach to discover new insights and relevant information regarding the perceptions of MHPs about their adult patients’ coping with ACEs during COVID-19 in Houston, Texas. The first author also created working definitions for each code to develop an initial codebook. Through this open coding approach, the first-level codes were generated to assess each interview transcript in detail. Then, the first author used the constant comparative method to evaluate and compare the data related to each first-level code to alter existing code definitions and develop second-level analytic codes to include new data discovered in the interview transcripts. The coding process continued and was reviewed continuously to develop the codebook, which included the list of primary codes, working definitions of the primary codes, and examples of the data with representative quotes from the transcripts in relation to the primary codes. Once all the transcripts were coded, the coded texts were grouped by similar codes, including the first- and second-level analytic codes from which relevant themes were identified through thematic analysis. Finally, the first and the second authors reviewed the relevant themes and the codebook consistently to maintain cohesion of the qualitative analysis process of the interviews (Tracy, 2020).

## Results

Table 2 demonstrates the characteristics of the interviewed MHPs (*N* = 10), including their self-identified gender, current occupation, and the type of organization through which they provide mental health services. Eighty percent of the MHPs who participated in the in-depth interviews were female, 40% were licensed social workers, and 70% were affiliated with a treatment/research/education center.

**TABLE 2:** Mental health provider (MHP) characteristics (*N* = 10).

Characteristics	n	(%)
**Gender**		
Male	2	20%
Female	8	80%
**Occupation**		
Licensed Psychologist	2	20%
Clinical Assistant Professor, Geriatric Psychiatrist	1	10%
Licensed Social Worker	4	40%
Licensed Professional Counselor	1	10%
Assistant Professor, Licensed Psychologist	2	20%
**Organization Type**		
Private Practice	2	20%
Treatment/Research/Education Center	7	70%
University	1	10%

Geographical region: Greater Houston Area, Texas.

### Qualitative results

Four major themes emerged from the MHP interviews regarding the phenomenon of how their adult patients were coping with their ACEs during the COVID-19 pandemic, including: (1) Maladaptive emotional dissonance and coping outlets during the pandemic, (2) Difficulties with social connectedness and significance of social support, (3) Heightened daily life stressors and coping with the ongoing disruption of the pandemic, and (4) Changing interactions with the mental health system. The themes and subthemes are presented in Table 3.

**TABLE 3:** Key themes and subthemes.

Theme 1: Maladaptive emotional dissonance and coping outlets during the pandemic
Subtheme 1a: Difficulties with emotional processing of historical ACE trauma
Subtheme 1b: Increased propensity of risky health behaviors
Subtheme 1c: Influence of religion in coping with ACEs

**Theme 2: Difficulties with social connectedness and significance of social support**

Subtheme 2a: Interpersonal difficulties with connecting to others
Subtheme 2b: Lack of trust socially due to historical ACE trauma
Subtheme 2c: Significance of positive social support and meaningful activities

**Theme 3: Heightened daily life stressors and coping with the ongoing disruption of the pandemic**

Subtheme 3a: Pandemic-related social isolation and avoidance contributing to worsening mental health
Subtheme 3b: Financial hardships and family related stress during the pandemic

**Theme 4: Changing interactions with the mental health system**

Subtheme 4a: Synergy of psychotherapeutic psychopharmacology and psychotherapy
Subtheme 4b: Increased usage of virtual telemedicine mental health appointments

### Maladaptive emotional dissonance and coping outlets during the pandemic

#### Difficulties with emotional processing of historical adverse childhood experience trauma

According to the interviewed MHPs, processing ACE trauma through healthy coping mechanisms is vital to live a healthier life. MHPs stated that many of their patients with ACEs tell themselves false narratives about their trauma repeatedly, creating additional barriers and stress for themselves due to the lack of trauma coping guidance when they were younger, which consequently precipitates the development of comorbid mental health disorders. A majority of MHPs during their interviews emphasized that the COVID-19 pandemic heightened the risks of poor mental health and promoted an unhealthy lifestyle among their patients with ACEs. Patients who were already struggling with processing their trauma at baseline have seen the stress from the pandemic accentuate their inability to successfully adapt healthy coping behaviors related to their historical trauma.

*Processing’s also helpful, cuz it really gets into the thinking, right? So many people are in their head telling themselves these false things over and over which, becomes habitual. So, it feels like it’s real and true to them. So, helping them really see, that maybe it’s not true*…*and then they come up with a more, a truer, more helpful thought and practice of doing that* [MHP 4, social worker].

…*a lot of it is similar to what we do when we’re trying to process trauma in itself, but just kind of going back to the basics, right. Tips and like wellbeing to checking their sleep hygiene, their routines during the day (guidance during COVID-19). Cause if they’re quarantining at home, then they’re don’t really have a schedule anymore. we talk to regular trauma patients in general is how do you cope with, you know, you’re still processing something really difficult, but how do we get back to the basics*…*Um*…*appetite*…*eating well, sleeping well, exercising*… [MHP 5, social worker].

#### Increased propensity of risky health behaviors

Several MHPs stated that most of their patients coped with their ACEs through self-destructive risky behaviors such as alcohol and substance abuse, performing risky sexual behaviors (i.e., having multiple sexual partners), suicide ideation, and attempted suicide due to the intense trauma they suffered during their childhood. Furthermore, the COVID-19 pandemic created an increased risk of these risky behaviors due to continuing disruption in their daily lives from the pandemic.

…*Some turns to alcohol and drugs to deal and to cope, some have been hospitalized like long-term hospitalization and are currently*…*out trying to work through those things*…*clients who’ve experienced sexual abuse as a child*…*acting out sexually with multiple sexual partners*… [MHP 1, psychologist, private practice].

*Substance use is definitely at the top of the list there because it’s a really effective short term avoidance coping strategy. You know, if you can drink or use drugs, then you’re not gonna be bothered by things that trigger your trauma history* [MHP 10, assistant professor and psychologist].

#### Influence of religion in coping with adverse childhood experiences

Mental health providers described how their adult patients with ACEs used religion both positively and negatively to cope with their ACEs. Some of the patients rely on their church community to have social support around them to cope with their ACEs positively. However, during the peak of the pandemic, individuals with ACEs who coped with their trauma by going to church for the communal support it provides, faced an upheaval of mandated social isolation policies of the pandemic which abruptly disrupted community in-person activities, and therefore taking away a critical venue that these individuals used to cope with their ACEs. For other patients with ACEs, religion has made it hard for these individuals to cope with their ACEs due to the social pressure placed by religious familial members for everyone to be perfect, and accordingly to be secretive about and not seek treatment for historical ACEs due to fear of societal stigma.

*I notice a lot of Spanish speaking patients, um, that normally would rely on like a church community and, um, being able to attend weekly services*…*that was a really big deal, not having that. So how do I adapt right. In, in the times of, of a pandemic, how do I still stay close with those people and cope? So*…*for my like immigrant minority population, I see religion as a, much bigger coping skill*… *And that’s something that provides you so much comfort and support and people around you that are supportive* [MHP 8, social worker].

*I definitely think that there is some of my clients who maybe higher religiosity*…*like there’s more of a tendency of families to like, okay, lock it down, don’t tell anybody*…*or being blamed for what they’ve been through*…*I think that has been really hard and sometimes religion has been part of the*…*traumatic events or*…*anything that makes it even harder*…*even more of like secretive or like, we can’t tell anybody. like they seem to have suffered longer, like before they end up getting mental health treatment, because*…*well, you’re not supposed to do that. Like you’re supposed to just to go to church, you’re supposed to like be part of the family, be in your community* [MHP 3, psychologist, private practice].

### Difficulties with social connectedness and significance of social support

#### Interpersonal difficulties with connecting to others

The majority of MHPs emphasized that the nuances of trauma due to ACEs have instigated renewed struggles to connect with others and develop and maintain healthy relationships. In addition, the COVID-19 pandemic heightened their stress to develop and stay intimate in their romantic relationships and maintaining existing relationships with family and friends due to the ongoing disruption of extreme isolation, burnout, and lack of social connectedness during the pandemic.

…*the ones that have been severely neglected or um, you know like*…*the ACE wasn’t that impactful at first. And then you notice later on saying. I don’t feel connected to people because I can never be intimate with somebody. And I never disclose information about myself. I always have to have this protective layer with me. And so, I feel lonely all the time*…*because of that, I feel depressed*… *I’ve never learned how to tolerate my emotions. when I’m stressed out, then I either eat my feelings out, drink my feelings out or*…*I just stay so busy*…*that I’m always exhausted because I can’t like find a way to just kind of relax* [MHP 5, social worker].

*Oh, it (COVID-19) just made it so much harder. So, if, if they had kids like trying to like situate childcare and, if you wanna talk about stress*…, *like not having your kids taken care of, plus you have to work, plus they have school, all of that stuff. Um, or it could be that some of these folks have elderly relatives that they’re trying to be a caretaker for plus kids plus everything else*…*I think the lockdowns made it worse in terms of romantic relationships*… [MHP 9, assistant professor and psychologist].

#### Lack of trust socially due to historical adverse childhood experience trauma

Mental health providers emphasized that the nuances of ACE trauma intrinsically led to severe mistrust of others faced by individuals with ACEs due to the abuse they have faced during their childhood; however, social trust was mentioned as a key attribute by MHPs for ACE patients to be able to successfully process their trauma. The COVID-19 pandemic made it difficult for individuals with ACEs to stay connected to their trusted individuals, especially in-person, which has consequently heightened their issues with mistrust of others; this has led to depression, hopelessness, and fluctuation of their negative emotions. Furthermore, the issues related to lack of trust among ACE patients relate to their own experiences suffering from trauma instigated by people they used to trust in their lives. The MHPs highlighted those issues related to mistrust have caused problems among their patients with ACEs to develop healthy relationships and maintaining these relationships during the pandemic.

…*I see a lot of the clients leading to a lot of difficulties in adulthood, and just the mistrust of most people around them*…*I think with COVID just to spend so much time isolating, and some people are then like stuck at home with somebody who is continuing to abuse them or be very neglectful of their relationship*…*a lot of people go into some pretty significant depressive episodes. I would say that suicidality seem to fluctuate*…*partially because it would seem like we were getting close and then when there would be another wave of COVID, then it was like and we’re back here. We’re doing this again*…*just incredibly crushing* [MHP 3, psychologist, private practice].

*Full on attachment issues*…*they can’t, trust somebody to build a relationship with and they can’t trust themselves to reveal their information. And so it’s like this constant, like I cannot connect. So, it’s just with like self-esteem because of that and power and control and everybody’s against me and nobody’s for me and just not able to have very positive interpersonal relationships* [MHP 5, social worker].

### Heightened daily life stressors and coping with the ongoing disruption of the pandemic

#### Pandemic-related social isolation and avoidance contributing to worsening mental health

The COVID-19 pandemic led to additional stress among adult patients with ACEs due to COVID-19-related social distancing guidelines, which exacerbated the avoidance of social situations and consequently mitigated healthy coping outlets for processing their ACE symptoms. This then culminated in ACE patients, per the interviewed MHPs, suffering more symptoms from co-occurring mental health disorders such as anxiety, depression, and PTSD.

*People who live by themselves and are completely isolated or*…*PTSD all based on avoidance. Right? So, the first thing they’re gonna do is they’re gonna isolate*…*from people, and then you have this beautiful excuse behind you that says, COVID, I’m gonna use this as an excuse to continue isolating myself and detaching from other people*…*and so then that kinda takes them even deeper on their depression and inability to like actually expose themselves to things that would be helpful for them*… [MHP 5, social worker].

*I will say people’s anxieties in terms of depression, like all of that worsened in COVID. And so, people did talk about, um, feeling isolated, right? Not being able to connect with other people. Um, and because they’re at home and stuck at home, their symptoms are worsening and not knowing how do I do this* [MHP 7, professional counselor].

#### Financial hardships and family related stress during the pandemic

Mental health providers emphasized how the pandemic-related financial hardships due to job loss, family related stressors such as loss of a closed one due to COVID-19, taking care of the kids with virtual schooling, and dealing with their own daily life stressors have made it extremely hard for their patients to cope with their ACEs currently. In addition, some MHPs stated that some of their patients also had difficulties to afford health insurance, limiting their access to receive health services for their ACEs, and a noticeable increased prevalence and severity of mental health issues due to the unparalleled obstruction caused by the pandemic.

*So, a lot of parents who don’t know how to cope with what’s going on*…*then we’re seeing the effects on the children who are just not knowing how to have any type of emotional regulation. They just don’t know how to self-regulate in schools and the amount of stress that they put on the parents causing parents stress out and then take it out on their kids. So*…*a ton of emotional abuse, a ton of parents like leading for substance use is a way to cope and then taking it out on the kids*…*So yeah. COVID is really messing with people’s mind* [MHP 5, social worker].

*People who had partners or they themselves lost their jobs, feeling much more stressed. Right. Not knowing how their gonna pay bills or what they’re gonna do next*…*probably very early in the pandemic had a patient have to drop out of treatment because their partner had like lost their job. And with that loss of insurance or at the time, I don’t think we had grants to cover their services but say that’s definitely a barrier* [MHP 7, professional counselor].

#### Significance of positive social support and meaningful activities

The MHPs stressed that individuals with ACEs should have trusted individuals from their families, social, or community groups to help them cope with their ACEs. The COVID-19 pandemic made it extremely difficult for their ACEs patients to connect with their social support in-person. The MHPs stressed that positive and continued support from trusted family members, peers, and community organizations is crucial to help individuals cope with their ACEs so that they do not perform self-destructive behaviors that can lead to poor quality of life and early death.

*I think biggest thing is with ACE is just being able to have a social support, right. So*…*if therapy is something that is not for you*…*just knowing that you’re able to be connected with somebody else that can support you, I think that sense of belonging and connectedness, or if they attend like a church, any groups, any type of like profession of work, something that has people involved where*… *they’re like micro healing because of all these positive connections that they make with people, I think*…*it doesn’t ever cancel out what happened when you were a child, but it, it almost creates this*…*little balance of*…*a positive interaction* [MHP 5, social worker].

*I think having a healthy support system, that’s one thing, the other one is being able to recognize*…*through therapy*…*that what they experienced was not their fault or*…*because of traits that they possess. and then I think when you have those right*…*and you have that support that opens up so many doors, cuz you can then, focus on like whatever goals you have for yourself with that person going forward* [MHP 8, social worker].

### Changing interactions with the mental health system

#### Synergy of psychotherapeutic psychopharmacology and psychotherapy

The MHPs stated that taking a combination of psychotherapy and psychotropic medications reduced stress levels and symptoms related to ACEs. Typically, individuals with ACEs tend to suffer from comorbid chronic mental health conditions such as severe depression, anxiety, PTSD, and borderline personality disorder; therefore, MHPs stated that it is vital that individuals with ACEs take psychotropic medications to regulate their emotions while concurrently receiving psychotherapy treatments, to optimize a holistic approach to helping ACE patient overcome their emotional challenges and mental health disorders in a structured manner.

*So, when it comes to medications, we always kind of, have to have a specific and particular kind of diagnosis for it. if a person has major depressive disorder, generalized anxiety disorder, post-traumatic stress symptoms, bipolar illness*…*we kind of treat those*…*symptoms that we see*…*I think specifically when it comes to kind of coping with ACEs and COVID-19, I think*…*it can just be plain old therapy, which is kind of just supportive, listening, and being in that environment with some regularity. And that sometimes it is kind of a more discreetly focused kind of cognitive-behavioral therapy*…*for anxiety or depressive symptoms for insomnia, or kind of coping with kind of chronic diseases like pain are very effective* [MHP 2, clinical assistant professor and geriatric psychiatrist].

…*DBT to kind of get the skills to be an under behavioral control, in order to be able to treat the trauma, and then doing a DBT, prolonged exposure. Um, those have probably been the main, treatments*… *I’ve definitely found that medication*… *I’m thinking of depression sometimes just getting someone on an antidepressant*…*that can give us like just enough space so that we can do behavioral activation* [MHP 3, psychologist, private practice].

#### Increased usage of virtual telemedicine mental health appointments

A majority of the interviewed MHPs reported that the utilization of tele-mental health services increased exponentially during the pandemic. Tele-mental health services improved accessibility to mental health services given social distancing guidelines, enabled MHPs to reach even more patients, while decreasing the social and intrapersonal barriers for ACE patients to seek care. However, MHPs mentioned that due to the drastic rise in tele-mental health services utilization, there were longer wait times for patients to get their appointments as a lot of mental health centers were fully booked up. Furthermore, the elderly population also had difficulties at first with using technology for their telehealth appointments, until they were provided technical assistance from MHPs. Ultimately, MHPs were able to provide a therapeutic avenue for adults with challenges related to historical ACEs exposures to seek treatment.

*We’ve been able to reach out to patients that are in the entire state of Texas. So, somebody who’s like living in Corpus and have only had access to the services that were around and that community now can access something in Houston and because they don’t have to drive all the way*…*it opened*…*so many more opportunities for them to access services* [MHP 5, social worker].

…*a waiting process, because a lot of the, uh, mental health community, mental health centers appear to be booked up and difficult to get in, get a slot and get started. And I think with just the accessibility has made it a little bit tougher to seek that treatment* [MHP 6, social worker].

*I think initially some of the older patients were maybe a little bit hesitant and it was a challenge for them cuz maybe they weren’t used to using app and downloading like zoom and using all these things*…*but once we were able to teach them and like get them used to it I think they were on board*… [MHP 7, professional counselor].

## Discussion

This study’s contribution to the literature is its focus on mental health providers (MHPs) who provide care for a diverse patient population of individuals with mental health conditions and ACEs, spanning all age, racial, and socioeconomic groups. This study provides contextual information generated from MHPs of the challenges that their patients with multiple ACEs have during an ongoing pandemic.

Compared to the general population with ACEs, the prevalence of exposure to at least one ACE among the health services populations have been reported to be higher by 58–88 and 18–38% for four or more ACEs, respectively (Riedl et al., 2020). Another study that assessed the correlates of ACEs among individuals with severe mood disorders reported that 50% of the sample reported three or more ACEs, indicating that health services samples have elevated rates of ACEs compared to the general population (Lu et al., 2008; Riedl et al., 2020). Studies have also shown that individuals exposed to multiple ACEs are more likely to commit suicide, engage in risky behaviors, and shorten their lifespan due to poor physical and mental health outcomes (Mersky et al., 2013; Hughes et al., 2017; Ross et al., 2020). The co-occurring substance use disorders, including alcohol and tobacco use, have caused additional burdens among individuals to cope with their multiple ACEs (Hughes et al., 2017). Thus, it is imperative that clinical practices must be informed and trained to prevent, detect, and reduce the effect of multiple ACEs and improve health outcomes by incorporating resilience-building and trauma-informed services for individuals with multiple ACEs (Hughes et al., 2017). Furthermore, most individuals with ACEs can improve their symptoms and health by being exposed to sources of resilience (Bellis et al., 2022). Resilience is developed intrapersonally through maintaining positive cognitive and emotional processes, interpersonally through enhanced access to social support, and developing connectedness to the local communities (Bellis et al., 2022). These were all themes echoed by MHPs in their interviews, with many of these factors being adversely impacted by the current pandemic’s social distancing guidelines disrupting traditional venues of healthy coping used by ACE patients. It is notable that resilience focuses on mature intra-personal coping in the midst of life stressors; yet its development and refinement are heavily influenced by and dependent upon healthy inter-personal social influences (Ross et al., 2020). Indeed, high levels of resilience mitigate the detrimental psychosocial and physical health effects of ACE exposures, but this resilience is dependent upon healthy community and relational resources for the individual (Ross et al., 2020).

The social isolation and mandates of the pandemic have reinforced negative cognitive and emotional dissonance in individuals with ACEs; furthermore, they have instigated systemic and structural barriers, as evidenced in our qualitative study, to interpersonal social support that can enhance resilience in patients with ACEs (Bledsoe et al., 2021). The loss of venues for social connectivity, heightened sense of distrust of others, and potential repeated exposure to stresses domestically without escape (Bryant et al., 2020), in the midst of social distancing policies of the pandemic, have cumulatively curtailed resilience in ACE patients. In the context of a high-stress environment of the pandemic, there were fewer available outlets to engage in honing and developing healthy coping skills to deal with stress, as mentioned by our MHPs. Consequently, unhealthy behaviors were exacerbated, and this induced worsened symptoms from co-occurring mental health disorders such as anxiety, depression, and PTSD and increased the inability of patients with ACEs to cope with their ACE-related emotional health symptoms, as seen elsewhere (Sonu et al., 2021).

Given the accentuated interpersonal difficulties of connecting to and trusting others among ACE patients, intentional efforts by health providers of patients with ACEs should be to highlight available social resources as society re-opens. This includes being aware of and promoting the creation and growth of social support groups for individuals who have ACEs and encouraging individuals with ACEs to participate in community events that are of most interest to them (Bledsoe et al., 2021). For example, our study identified religion and faith as an important factor for patients of several of our MHPs, and therefore faith-based social support may be a vehicle to augment ACE patients’ resilience levels. This mitigation strategy would redevelop the intra-personal resilience that is necessary to mitigate the morbidity and mortality of ACE exposure on one’s health (Ross et al., 2020), which is particularly critical given the context of the ongoing COVID-19 pandemic.

Furthermore, from the healthcare systems aspect, our study overall found that MHPs have felt that their use of digital provision of healthcare care through virtual telemedicine platforms has increased the accessibility of mental health services for patients with ACEs. The MHPs felt that the incorporation of telemedicine services has decreased the barriers for individuals with ACEs who desire to receive care. Barriers related to transportation and anxiety of going to a clinic setting were abated by doing a health visit from the comfort, convenience, and security of their home. Initially, the Veterans Affairs (VA) system implemented telehealth services in 2002 for its patients nationally, including in the Houston area, although uptake has been slow but progressively increasing (Abel et al., 2018). According to Der-Martirosian et al. (2020), in the Houston, Texas VA system, the uptake of telehealth services, encompassing telepsychiatry and telehealth programming for primary care visits, was about 20% of all outpatient encounters in this system from 2016 to 2018. Der-Martirosian et al. (2020) noted that mental health was the second most common type of clinical service provided by telehealth services after primary care visits in the Houston VA system. Notably, Der-Martirosian et al. (2020) discovered that public health emergencies and disasters could impact the uptake of telehealth programming provided to VA patients, as was seen during Hurricane Harvey, where the proportion of all patient encounters conducted *via* telehealth expanded from 20% at baseline to 55% 6 days post-Hurricane. Although the share of telehealth visits then regressed to the baseline about 2 weeks after the Hurricane, this short jump foreshadowed the ability of telehealth services to be a critical source of extending care to vulnerable patient populations during times of emergent crises, as was seen in the pandemic. The positive reception of the use of telehealth to fill in gaps of service to local Houston patients to provide medical care during the context of the uncertainty and stress during Hurricane Harvey in 2017 may have influenced local providers to be receptive to embracing telehealth services during the uncertainty of the current pandemic public health emergency. The effects of the pandemic have been more long-lasting than that of a hurricane, and accordingly, it is expected that the benefits and convenience of telehealth services will continue to contribute to a new baseline in the share of outpatient encounters that use telehealth as its modality for delivery. However, the transition to telepsychiatry is not entirely positive from the providers’ perspective. Some negative perceptions of telepsychiatry include the decrease in the formality of the encounter, including increasing the chances of disruptions during the encounter while at home, which can impair patient-provider rapport, and a perceived loss of work-life balance as the space and time separation between home and work can become blurred for the provider (Sasangohar et al., 2020).

At a systemic level, the role of telemedicine in behavioral health is particularly salient in Texas, with its status of having the most rural-residing residents of all states, and the fact that the vast majority of MHPs in Texas work in just 5 out of the state’s 254 counties (Tarlow et al., 2020). In a longitudinal study on Texas individuals with depression, self-reported severe disruptions to daily routines and disruptions in access to mental health treatment in having appointments canceled or postponed led to greater depressive and anxiety symptoms in the early pandemic period (May–August 2020) relative to pre-pandemic levels (Czysz et al., 2021). This suggests the critical role played by having consistency at least in MHP outreach and attention, for the persistent recovery and psychological maintenance of individuals with mental health conditions, such as ACEs. Telemedicine, therefore, remains a prominent strategy for MHPs to increase their reach to individuals with ACEs, enabling a form of stability in the consistent provisioning of mental health care in an uncertain world, while also serving as a less stress-inducing milieu for individuals with ACEs, as they need not leave the familiar environs of their home.

In contrast to the interviews in which the reception to telepsychiatry services was universally positive, it should be noted that telepsychiatry services have also exacerbated inaccessibility, especially for those with low digital literacy, which required MHPs to provide additional training and education to their patients with low digital literacy on how to access their telepsychiatry appointments remotely. Therefore, those who were less likely and less compliant to care pre-pandemic are not engaging in care post-pandemic (Nouri et al., 2020). In addition, our MHPs highlighted that the ongoing disruption of the pandemic created longer wait times for scheduling telehealth appointments due to the mental health centers and private practices being overbooked. The longer wait times have caused immense difficulties among their patients to cope with ACEs, leading to worsened mental health outcomes.

At the provider level, Fegert et al. (2020) suggests that there is a long road back to “normalcy” after this pandemic and that providers need better mechanisms for ACE screening, particularly in adults and youth that may have exposure to ACEs, in order to develop and initiate personalized treatment, and connect them to social connectedness programs. Additionally, MHPs should enhance their therapeutic rapport with individuals with ACEs to a population who, due to trauma, have- as mentioned by our MHPs- inherent social distrust of others and resort to self-isolation (DeCandia and Guarino, 2015; Bevilacqua et al., 2021).

Our MHPs noted how the pandemic has exacerbated the difficulties with insurance and increased financial uncertainty, adding stress to ACE patients, and serving as a poignant barrier to their long-term recovery as receiving access to care is now harder. Accordingly, the intersectionality of social determinants of health on the engagement of health behaviors, seeking and complying with mental and medical healthcare, and health trajectories of patients with ACEs should be explored to provide a platform of equity advancement (Gutschow et al., 2021). Amid the pandemic, clinics should rebuild and strengthen links to local community resources, with social services referrals to target the social determinants of health barriers that may preclude successful access to and outcomes of healthcare treatment (Lee and Forkey, 2022). This will consequently promote resilience in patients with ACEs. The paradigm shift in healthcare should be not for providers to prescribe and rely on pharmacological interventions to magically “cure” a chronic condition like ACE, but rather to focus on trauma-informed practices to empower patients themselves to develop, hone, and mature their intra-personal resilience to adequately augment the psychosocial and physical health trajectories of individuals with ACEs (Lee and Forkey, 2022). Our MHPs discussed the synergy seen when combining psychotherapeutic techniques with psychopharmacology for patients with ACEs who also have comorbid mental health conditions. This is critical because the ability to fully engage and derive benefit from psychotherapy techniques designed to enhance one’s resilience and coping techniques is predicated upon the stabilization of acute mental health symptoms like depression or anxiety, which typically occur through the medication optimization (Fegert et al., 2020; Lee and Forkey, 2022). In other words, psychopharmacological medications synergistically work with psychotherapy modalities to produce optimal clinical prognosis in individuals with ACEs. This duality of needing both psychotherapeutic and psychopharmacological modalities of treatment needs to be the model of care provided to ACE patients, even with the transition to tele-psychiatry services due to the pandemic, to holistically address their mental health, cognitive, and emotional needs.

Although concerns have been raised about the quality of care and diagnosis provided to patients due to the inability to perform physical exams directly with telehealth services, the reliance in psychiatry on verbal and clinical observations will likely have no to little impact on the quality of care provided to mental health patients with telepsychiatry visits (Solomon and Soares, 2020). Indeed, the use of telehealth services with the pandemic may facilitate the ability of MHPs who may specialize in just one modality of treatment for mental health patients (e.g., therapy) to refer them to other MHPs who can handle synergistically the other modality of treatment (e.g., pharmacological). Telepsychiatry can also enhance interdisciplinary communication between providers to ensure coordinated care is occurring for the mental health patient (Solomon and Soares, 2020). Unlike traditional in-person visits where visits to separate MHPs may be needed and therefore travel costs and barriers to visit two separate providers incurred by the ACE patient, telepsychiatry allows ACE patients to access all needed services from the convenience of home.

### Limitations

The limitations of this study rest with its limited sample size which can mitigate its external validity to other providers and patients with ACEs in different contexts. Additionally, the study interviewed providers to get indirect information about the impact of the pandemic on patients with ACEs, rather than interviewing patients with ACEs. This was done because of the ethical difficulty of interviewing patients with ACEs and to enhance the efficiency of the general contextual themes generated in this qualitative analysis by speaking with providers who each care for hundreds of patients with ACEs, rather than interviewing patients with ACEs individually. Additionally, individuals with mental health conditions are considered a particularly vulnerable group for research (Keogh and Daly, 2009), and therefore merit enhanced safeguards in considering whether to include them in qualitative interviews that can cause the exacerbation of behavioral symptoms and cognitive dissonance. The authors ultimately decided to err on the side of caution and interview only MHPs for this study. Indeed, qualitative interviews involving individuals with mental health conditions tend to have an insufficient justification of ethical principles meant to protect this vulnerable group, with issues related to coerced informed consent and biased recruitment (Carlsson et al., 2017). However, as we envision this study being a foundational study, in future research we plan to incorporate the perspectives of individuals with ACEs from both those seeking care and those not seeking care to ascertain their perspectives and juxtapose their insight from that of the providers of this study. The thematic analysis generated from this study indirectly includes, from the vantage point of our interviewed MHPs, interactions with ethnic minority patients to detail their unique challenges, as they have historically been neglected in ACE studies, yet tend to have a greater propensity of receiving ACEs and suffer from their detrimental effects due to historical and current oppression and neglect (Merrick et al., 2018; Sonu et al., 2021). Future research should focus primarily on ethnic minority and socioeconomically disadvantaged individuals with ACEs as their perspectives and therefore needs have been neglected in the literature to date.

Further, by only interviewing MHPs, we by default only gathered qualitative thematic elements of patients with ACEs who *actively* seek and thus received care for their ACEs and mental health issues. In general, men, minorities, and older adults are less likely to utilize mental health services and therefore seek professional assistance from MHPs for their behavioral health conditions, such as ACEs (Tarlow et al., 2020). Therefore, our MHPs would not be able to provide as much perspective on these individuals who did not seek care. Many individuals with ACEs have seen this pandemic mitigate their trust in and therefore desire to engage in treatment for their symptoms (Claypool and de Peralta, 2021; Bellis et al., 2022). Exposures to the upheaval of daily life from the pandemic can trigger individuals with a longitudinal history of ACEs to the point where they, in the act of self-perseveration and coping to a perceived stressor and existential threat, will seek immediate self-gratification and lose sight of the altruistic perspective of needing to engage in behaviors to protect others (Meldrum et al., 2020; Bevilacqua et al., 2021). Instead, they accentuate their perceived victimization and trauma-instigated immense distrust of authority figures, including toward health professionals that they feel are exacerbating the upheaval experience of the pandemic (Bellis et al., 2022). Therefore, even if services have been expanded due to use of telehealth, there is also a heightened intrinsic lack of motivation to seek care among some patients with ACEs, despite having active medical or psychiatric comorbid disorders that necessitate professional follow-up (Sonu et al., 2021). Ultimately, it has been noted nationwide there is an increasing propensity for lapses of care in individuals with ACEs, who also face higher susceptibility to and morbidity to COVID-19 compared to the general population (Földi et al., 2020; Huang et al., 2020; Bellis et al., 2022). Accordingly, the themes generated in this study may not be reflective of individuals with ACEs who never seek medical care with a MHP.

## Conclusion

Through our qualitative interviews with mental health providers (MHPs) who provide care for a diverse patient population of individuals with ACEs, we discovered that the pandemic has instigated additional barriers and opportunities for individuals living with ACEs. Our interviewed MHPs revealed that there was intrapersonal maladaptive cognitive and emotional dissonance, intrapersonal discovery of new coping outlets that were risky (e.g., drug use) and beneficial (e.g., faith), persistent and accentuated difficulties with social support, sociocultural stressors during a pandemic, and changing interactions with the mental health system. Overall, MHPs are attempting to empower their patients with ACEs to develop their own resilience, and to find outlets conducive to developing resilience such as strong social support, to enable their patients to continue their personal journey of processing their traumas. A resilience-promotion approach that targets all the socio-ecological levels, in addition to highlighting the synergy of psychotherapeutic and pharmacological management *via* tele-health modalities, may help inform best clinical practices in helping individuals with ACEs to continue receiving the care they deserve and need during a persistent pandemic and an uncertain future.

## Data availability statement

The original contributions presented in this study are included in the article/supplementary material, further inquiries can be directed to the corresponding author.

## Ethics statement

The studies involving human participants were reviewed and approved by the University of Texas Health Science Center at Houston. Written informed consent for participation was not required for this study in accordance with the national legislation and the institutional requirements.

## Author contributions

SC, PY, and CM contributed to the conception of this study and drafting and editing of the manuscript. SC designed the study, conducted the interviews, and analyzed the data. SC and PY reviewed the codebook. All authors contributed to the article and approved the submitted version.

## Footnotes

1
https://www.temi.com/
